# Tumor Microenvironment Profiles Reveal Distinct Therapy-Oriented Proteogenomic Characteristics in Colorectal Cancer

**DOI:** 10.3389/fbioe.2021.757378

**Published:** 2021-10-28

**Authors:** Nan Wang, Rongshui Wang, Xia Li, Zhentao Song, Lingbo Xia, Jue Wang, Li Zhang, Aiwen Wu, Zhiyong Ding

**Affiliations:** ^1^ Mills Institute for Personalized Cancer Care, Fynn Biotechnologies Ltd., Jinan, China; ^2^ Department of Oncology, Shandong Provincial Hospital Affiliated to Shandong First Medical University, Jinan, China; ^3^ Department of Pathology, Beijing Cancer Hospital, Beijing, China; ^4^ Key Laboratory of Carcinogenesis and Translational Research (Ministry of Education), Department of Gastrointestinal Surgery, Peking University, Beijing, China

**Keywords:** tumor microenvironment (TME), digital spatial profiling, spatial proteomics, spatial transcriptomics, immunotherapy, PD1 and PDL1, CRC (colorectal cancer)

## Abstract

Advances in immunotherapy have made an unprecedented leap in treating colorectal cancer (CRC). However, more effective therapeutic regimes need a deeper understanding of molecular architectures for precise patient stratification and therapeutic improvement. We profiled patients receiving neoadjuvant chemotherapy alone or in combination with immunotherapy (PD-1 checkpoint inhibitor) using Digital Spatial Profiler (DSP), a high-plex spatial proteogenomic technology. Compartmentalization-based high-plex profiling of both proteins and mRNAs revealed pronounced immune infiltration at tumor regions associated with immunotherapy treatment. The protein and the corresponding mRNA levels within the same selected regions of those patient samples correlate, indicating an overall concordance between the transcriptional and translational levels. An elevated expression of PD-L1 at both protein and the mRNA levels was discovered in the tumor compartment of immunotherapy-treated patients compared with chemo-treated patients, indicating potential prognostic biomarkers are explorable in a spatial manner at the local tumor microenvironment (TME). An elevated expression of PD-L1 was verified by immunohistochemistry. Other compartment-specific biomarkers were also differentially expressed between the tumor and stromal regions, indicating a dynamic interplay that can potentiate novel biomarker discovery from the TME perspectives. Simultaneously, a high-plex spatial profiling of protein and mRNA in the tumor microenvironment of colorectal cancer was performed.

## Introduction

As colorectal cancer is one of the leading causes of cancer deaths around the world, multiple clinical trials have proven the efficacy and rationale for immunotherapy in improving treatment outcomes for late-stage colorectal cancer (CRC), especially for those bearing genetic traits of mismatch-repair deficient (MMR-D) and/or microsatellite instability (MSI-H) (Golshani and Zhang, 2020; Siegel et al., 2018; Overman et al., 2017; PD-1 Inhibitor Bests Chemo for Colorectal Cancer, 2020). Notwithstanding the effort made toward MMR-D and/or MSI-H CRC, of more clinical importance, about 95% of CRC patients are MMR proficient (MMR-P) and/or microsatellite stable (MSS). Although clinical trials were underway with expectations to benefit a potential subset of MMR-P/MSS patients using PD-1 (programmed death-1) modulation alone or in combination with other targeted agents as well as radiation and chemotherapy, convincing data are still largely lacking (Bilgin et al., 2017; Arora and Mahalingam, 2018; Eng et al., 2019; Golshani and Zhang, 2020).

A profound understanding of the tumor microenvironment (TME) within heterogeneous tissues is demanded to identify effective biomarkers in the rim of immunotherapy for CRC. Despite tumor mutation load as the primary driver of microsatellite instability in CRC, other mechanisms do exist, such as the high expression level of PD-L1 (programmed death-ligand 1) and close association between tumors expressing PD-L1 or PD-L2 (programmed death-ligand 2) and immune infiltrates (Taube et al., 2014; Salem et al., 2018). PD-L1 expression was shown to correlate strongly with CD8 (cluster of differentiation 8) T-lymphocyte infiltration in CRC TME, and this phenomenon appears to be associated with microsatellite instability (Sudoyo et al., 2019). A previous work using single-cell RNA sequencing (scRNA-seq) revealed highly complex T-cell subclones and distinct functions within CRC. Specific clusters of TH1-like (T-helper 1-like) T-cell co-expressing *CXCL13* (Chemokine C-X-C motif ligand 13) and *BHLHE40* (Class E basic helix-loop-helix protein 40) were associated with only microsatellite-instable tumors and shared an increased level of *IGFLR1* (IGF-like family receptor 1) with CD8 exhausted T cells, indicating likely co-stimulatory mechanisms and biomarkers for MSI-H patients (Zhang et al., 2018). Focusing on myeloid cell populations in CRC, other groups discovered novel *SPP1* (secreted phosphoprotein 1) expressing tumor association macrophage (TAM) that could play critical roles in CRC tumorigenesis. This subpopulation exhibits a tighter association with cancer-associated fibroblast stressing the dynamic cross-talk within the TME (Zhang et al., 2020).

Indeed, several lines of evidence support the idea of incorporating spatial information for the biomarkers profiling in CRC. The density and location of CD3^+^ and CD8^*+*^ T cells, and GZMB+ (Granzyme B) and CD45RO + are strongly correlated with the overall survival (OS) of CRC based on the early tissue microarray data (Galon et al., 2006). More in-depth studies continued to support that the quantitative evaluation of cytotoxic and memory T cells in the tumor core regions, and invasive margin “immunoscore” served as a more powerful predictor of patient survival than MSI-H (Mlecnik et al., 2011; Mlecnik et al., 2016). In MSS patients, “immunoscore” was also shown as a prognostic factor for survival (Nosho et al., 2010). Upon large-scale internal cross-center validation to prove the “immunoscore” as a parameter for prognosis in stage I-III CRC, the European Society for Medical Oncology has historically approved to consider the “immunoscore” as a prognostic factor to assist TNM scoring in stage I–III patients (Mlecnik et al., 2018; Argiles et al., 2020). Another study investigated the combinational power of PD-L1 expression and extracellular mucin percentage in predicting clinical outcomes (Llosa et al., 2019). To set a spatial phenotype in CRC, researchers employed 56-plex proteomic spatial technologies at the single-cell level to explore specific cell-type–oriented pathological architectures and defined cell population–based neighborhoods. This study proved that tertiary lymphoid structures (TLSs) defined by a high density of CD3/CD4 T cells, B cells, CD163 macrophage, and CD4/CD45RO T cells are associated with better clinical outcomes for Crohn’s-like reaction (CLR) phenotype, whereas PD-1/CD4 expression T cells in granulocyte defined neighborhood positively correlated with survival in diffuse inflammatory infiltration (DII) subtype of CRC (Schurch et al., 2020). Those together underpin the fundamental concept of spatial-assisted high-plex profiling in CRC biomarker discovery and validation.

Understanding the complex molecular profiles of CRC in high-plexity requires advanced analytical tools. Among those, linking spatial information with high-dimensional data at molecular levels has already started elucidating novel mechanisms for immunotherapy in CRC. Herein, we used Digital Spatial Profiler (DSP), a recently emerged technology for high-plex proteogenomic characterization of the TME, to explore MSI-L CRC patients receiving neoadjuvant chemotherapy alone or in combination with a PD-1 checkpoint inhibitor (Merritt et al., 2020; Wang et al., 2021a) in a protein–mRNA–coordinated manner.

## Results

### Validation of DSP on Colorectal Cancer Tissues

**FIGURE 1:**
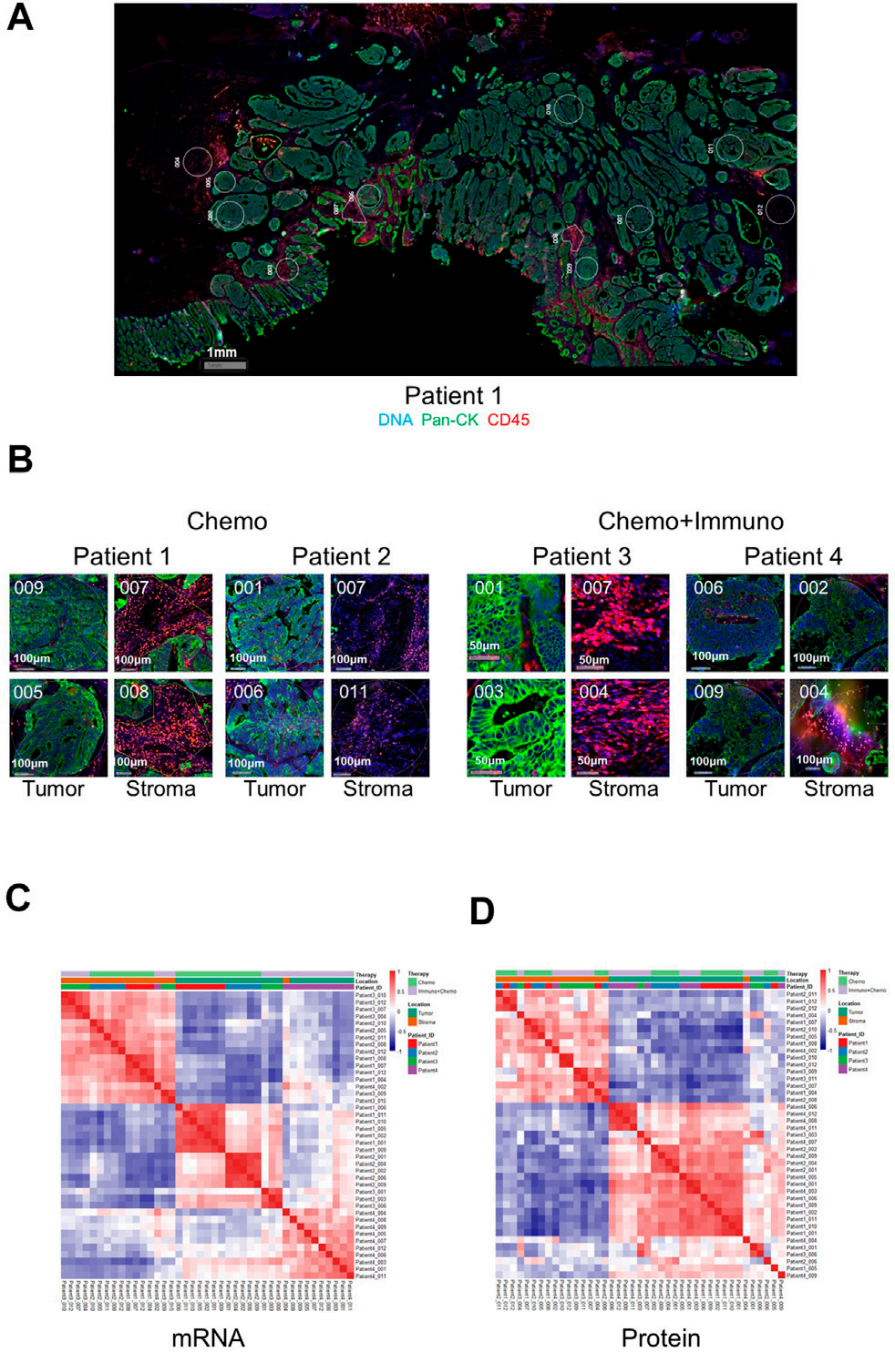
Representative full scan and regions of interest (ROIs) from patients and ROI-based correlation matrices on 84-plex RNA and 40-plex proteins. **(A)** Representative full-scan image for patient 1. **(B)** Representative ROI images for all patients. For every patient, 12 ROIs were drawn, and four representative ROIs (2 tumors and 2 stroma) are shown with tricolor fluorescence labeling (blue: SYTO13, green: Pan-cytokeratin, Pan-CK, red: CD45) at 20x magnification (patients 1 and 2: chemotherapy only, patients 3 and 4: chemotherapy + immunotherapy). For correlation matrices, ROIs are grouped based on treatment options, ROI location (tumor or stroma), and patient IDs. **(C)** mRNA-based expression correlation matrix shows separate stroma and tumor ROI clusters, and separate therapy-dependent ROI clusters in tumor regions. QC-failed and normal epithelium ROIs were excluded. **(D)** Protein-based expression correlation matrix shows separate stroma and tumor ROI clusters. QC-failed and normal epithelium ROIs were excluded.

We first validated the DSP technology by exploring gene-wise and ROI-wise association across all samples/genes. We selected tumor and stroma ROIs, given their expected distinct expression characteristics. The representative images of full scan and ROIs from either tumor or stroma are shown in Figure 1A. Representative ROIs from patients who received either chemotherapy only or combined with immunotherapy are shown in Figure 1B. Additional full scan images and ROIs are shown in Supplementary Figure S1. As expected, based on the expression profiles of 84 genes at the transcriptional level and 40 at the protein level in all ROIs, tumor and stromal regions showed strong region-specific expression patterns, regardless of individual tissue characters and different treatments (Figures 1C,D). The mRNA data from tumor ROIs also showed a therapy-differentiated expression pattern. A clear separation of patient groups with different neoadjuvant therapies by mRNA expression in tumor but not stroma ROIs suggests a potential immune signature in the tumor-enriched TME compartments (Figure 1C). Protein expression did not separate patient groups potentially due to the limited ROI numbers and protein targets in this study (Figure 1D). Also, the RNA or protein data for the 17 genes in common between 84-plex RNA and 40-plex protein panels did not separate patients according to different treatments (Supplementary Figures S2A,B).

For between-gene association analysis, common epithelial cell markers such as AKT1, Cytokeratin, EPCAM, Ki-67, and S6 showed a strong association at both the RNA and protein levels (Supplementary Figure S2C,D). At the protein level, the data showed a strong co-expression pattern between the T-cell markers (CD3, CD4, and CD8) and the myeloid-derived cell markers (CD14 and CD163). They are also significantly associated with the total immune cell markers (CD45/CD45RO/HLA-DR) and to a lesser extent with the B-cell markers (CD20), indicating an overall consistency of those markers being co-expressed within particular microenvironments (Supplementary Figure S2D).

### Region-Defined Individual Molecular Characteristics of the CRC Patients

**FIGURE 2:**
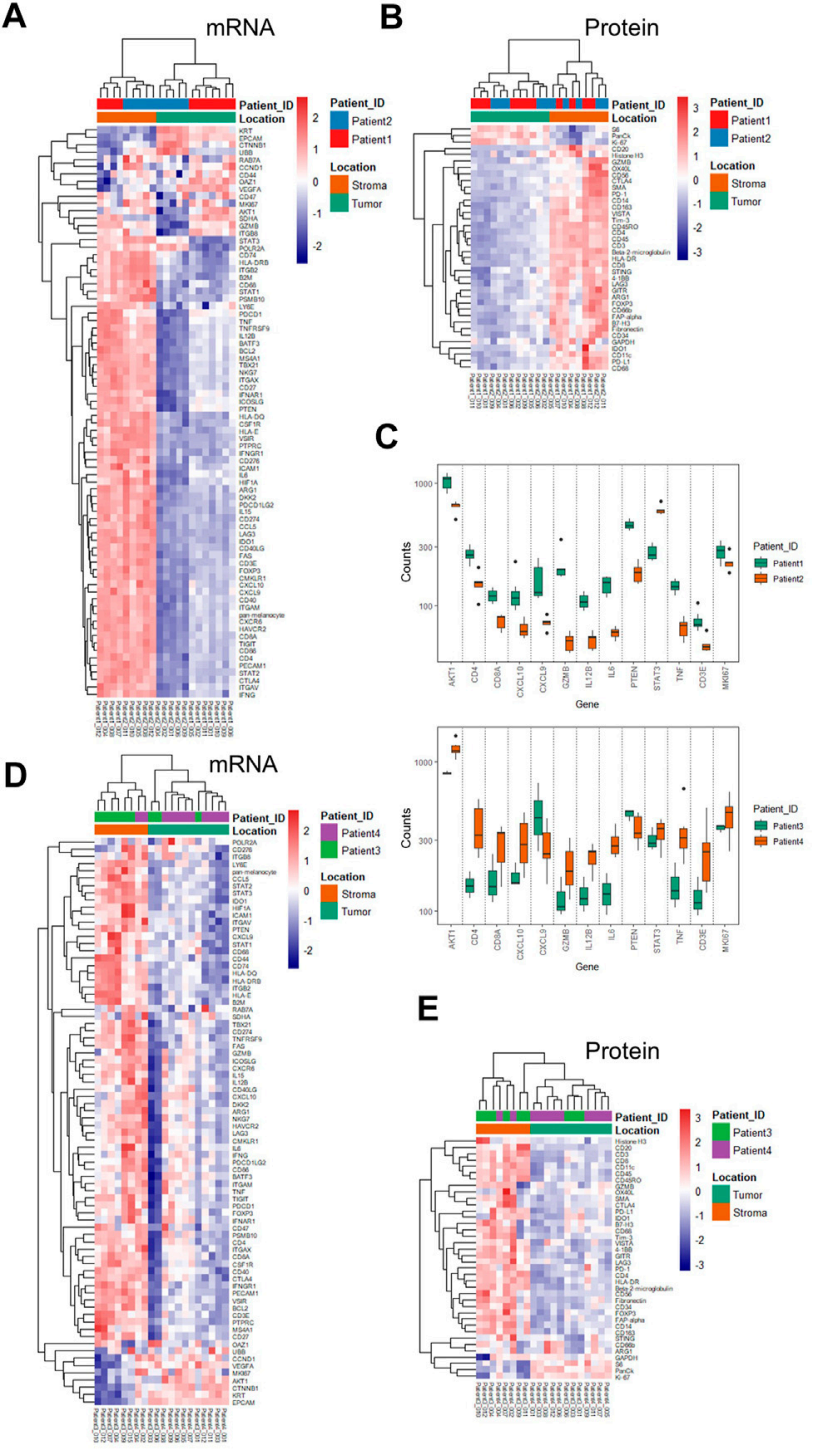
Unsupervised hierarchical clustering of mRNA and protein for ROIs from chemotherapy- and combinational therapy–treated patients and differentially expressed genes. **(A)** mRNA-based clustering of tumor and stroma ROIs of chemotherapy-treated patients 1 and 2. **(B)** Protein-based clustering of tumor and stroma ROIs of chemotherapy-treated patients 1 and 2. **(C)** Differential mRNA expression in the tumor ROIs between patient 1 and patient 2 (*p*-values were adjusted, and the cut-off was set to 0.05). **(D)** mRNA-based clustering of tumor and stroma ROIs of combinational therapy–treated patients 3 and 4. **(E)** Protein-based clustering of tumor and stroma ROIs of combinational therapy–treated patients 3 and 4. Color bars are grouped based on ROI categories (tumor/stroma) and patient IDs, respectively.

Based on the ROI selection strategy of the TME regions, we compared the tumor-surrounding stromal and tumor epithelial areas (ROIs) within or between the treatment groups. Chemotherapy patients (patients 1 and 2) showed a generally minimal immune marker expression at tumor-enriched regions at the RNA and protein levels, indicating low tumor-infiltrating lymphocytes (TILs) (Figures 2A,B). All immune-related cells resided in the surrounding stroma, with an apparent exclusion from tumors demonstrated by the overall low expression of the immune markers (Figures 2A,B). The RNA expression within the tumor-enriched regions revealed distinct expression profiling between these two patients, whereas those same sets of markers showed minimal differences within the stromal compartments (Figure 2C and Supplementary Tables S4, S5). Compared with patient 2, patient 1 showed higher levels of *GZMB*, *TNF* (tumor necrosis factor), *IL12B* (interleukin 12B), *IL6* (interleukin 6), *CD8A*, *CD3E*, *CD4*, and *CXCL9/10*, suggesting a more active status of immune infiltration. Patient 1 also showed higher levels of *PTEN* (phosphatase and tensin homolog), *AKT1*, and *Ki67*. Patient 2 showed an increased *STAT3* (signaling transducer and activator of transcription 3) (Figure 2C), likely due to the primary tumor difference or adaptation to the treatment. The differential expression above could only be discovered when the tumor-enriched regions and stroma regions were explored separately (Figure 2C, and Supplementary Tables S4, S5 for the entire gene lists). When comparing the 17 common genes, mRNA difference was not captured at the protein level in the same ROI profiling, emphasizing the importance of proteomics data in complementing transcriptomic data to stratify patients.

As expected, combinational therapy (chemo/immuno) induced various immune infiltration methods into the tumor regions manifested by an elevated expression of many immune markers, including *CD4*, *CD68*, *GZMB*, *CD40LG* (CD40 ligand), *CD86*, *CD276*, and *LAG3* (lymphocyte-activation gene 3) at the mRNA level in multiple tumor ROIs (Figure 2D). Chemokines and cytokines, such as *CXCL10* and *IL15*, *IL12B*, and *IL6*, and the receptor *CXCR6* (C-X-C chemokine receptor type 6), also increased at RNA levels. Concomitant increases of PD-L1, IDO1 (indoleamine-pyrrole 2,3-dioxygenase), OX40 (tumor necrosis factor receptor superfamily member 4), CD66b, and CD68 at the protein level were also observed (Figure 2E compared to Figure 2B).

**FIGURE 3:**
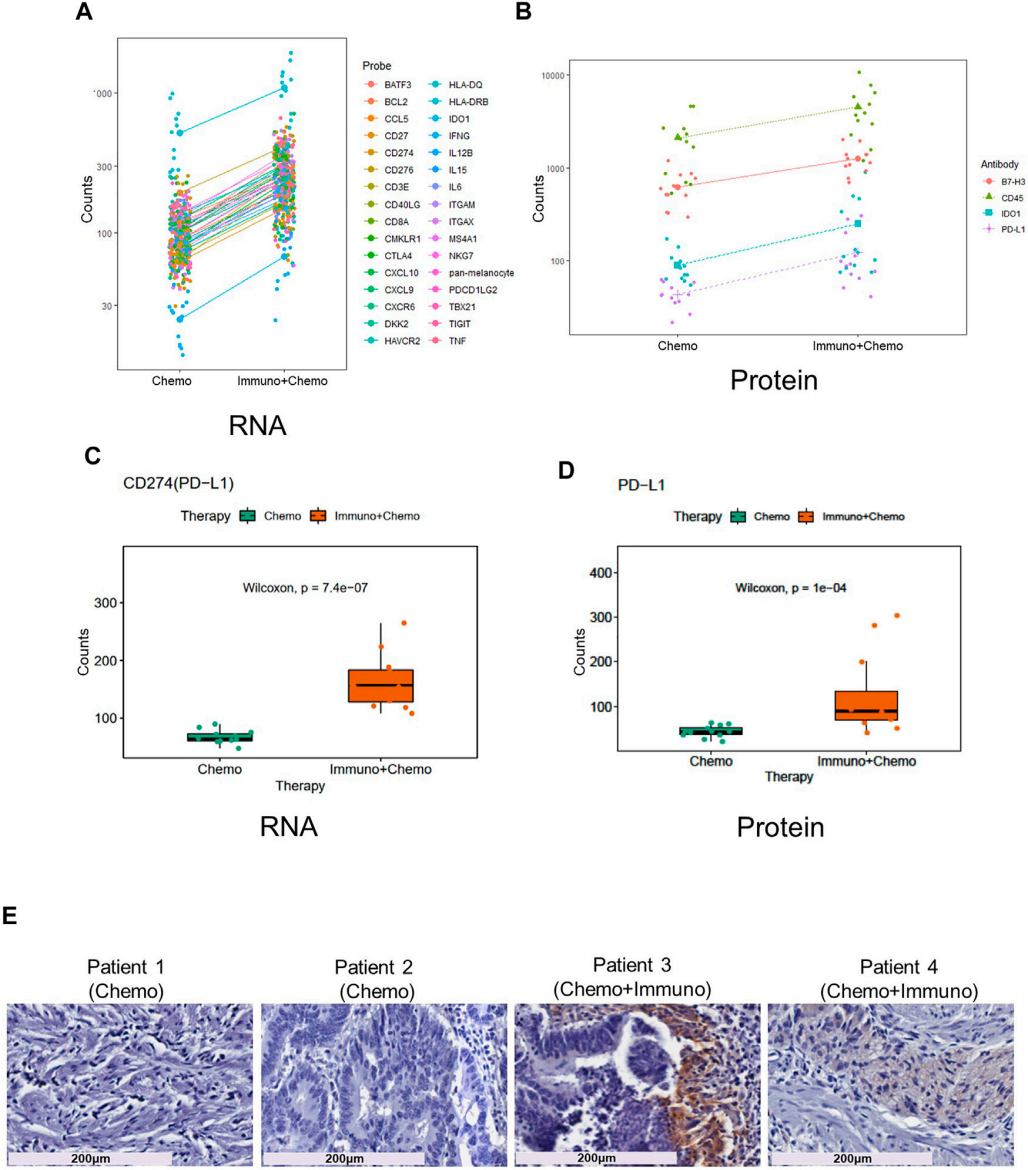
Increased immune response and elevated PD-L1 expression in combinational therapy–treated patients within the tumor ROIs. Line charts of differentially expressed mRNA **(A)** and protein **(B)** within the tumor regions between the treatment groups. Y-axes are untransformed expression raw count values of individual probes. Boxplots of PD-L1 mRNA **(C)** and protein **(D)** expression in ROIs of chemotherapy- and combinational therapy–treated groups (the Mann–Whitney test *p* < 0.05, non-adjusted). **(E)** PD-L1 IHC staining of patient tissues receiving chemotherapy or combinational therapies. Magnification is shown at 20x (scale bar =200 μm).

**FIGURE 4:**
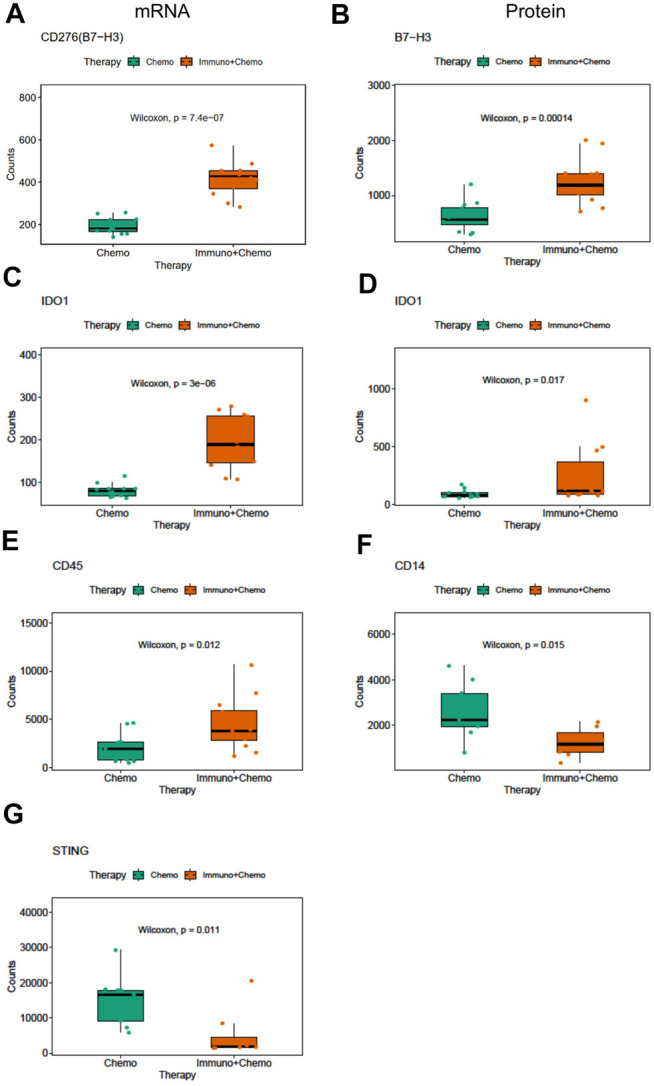
Differentially expressed mRNA and protein markers in tumor or stroma ROIs of chemotherapy or combinational therapy patients. Boxplots of CD276 mRNA **(A)** and protein **(B)** expression in the tumor ROIs of chemotherapy or combinational therapy patients (the Mann–Whitney test p < 0.05, non-adjusted). Boxplots of IDO1 mRNA **(C)** and protein **(D)** expression in the tumor ROIs of chemotherapy or combinational therapy patients (the Mann–Whitney test *p* < 0.05, non-adjusted). Boxplots of *CD45* **(E)**, *CD14* **(F)**, and *STING* **(G)** expression in the stroma ROIs of chemotherapy or combinational therapy patients (the Mann–Whitney test *p* < 0.05, non-adjusted).

We then compared inter-treatment group expression profiles and observed an overall high expression of immune markers at both the RNA and protein levels in the combinational treatment group, suggesting induction of immune infiltration (Figures 3A,B). As expected, we observed a significant (*p* < 0.001) tumor-specific increase of PD-L1 expression at both the mRNA and protein levels for patients receiving combinational therapy (Figures 3C,D). We validated this finding by conventional immunohistochemistry (IHC), where we observed an increased expression of PD-L1 in combination therapy–treated patients reconfirming the DSP data (Figure 3E). Another biomarker, B7-H3 (CD276), also increased at the mRNA and protein levels in the tumor regions of combination therapy–treated patients (Figures 4A,B). The IDO1 expression was also up-regulated markedly at both the mRNA and protein levels within the tumor regions of combination therapy patients (Figures 4C,D). Of note, CD45 only increased at the protein level but not at the mRNA level (Figure 4E), suggesting the need to evaluate the TME at both the omic levels. In addition, stromal ROI profiling identified an increased protein expression of STING (stimulator of interferon response CGAMP interactor 1) and CD14 in the chemotherapy group, a sign of immune exclusion in the presence of the chemo agent alone (Figures 4F,G).

**FIGURE 5:**
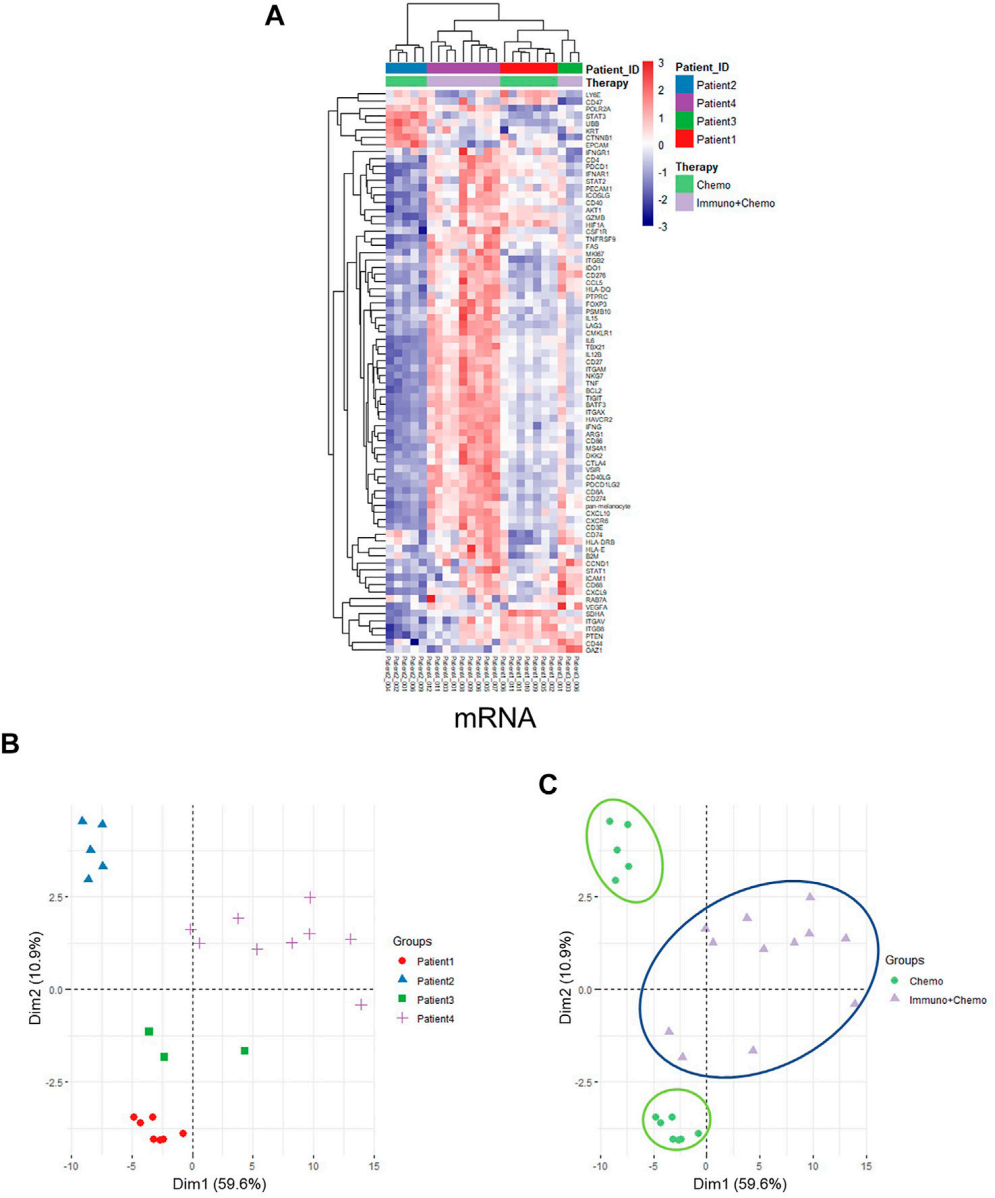
Unsupervised hierarchical clustering and PCA of tumor regions based on mRNA expression. **(A)** Unsupervised hierarchical clustering of mRNA expression in the tumor ROIs of chemotherapy or combinational therapy patients. ROIs are grouped based on treatment options and patient IDs. **(B,C)** PCA plots of mRNA expression in the tumor ROIs grouped by patients **(B)** or therapies **(C)**, respectively.

**FIGURE 6:**
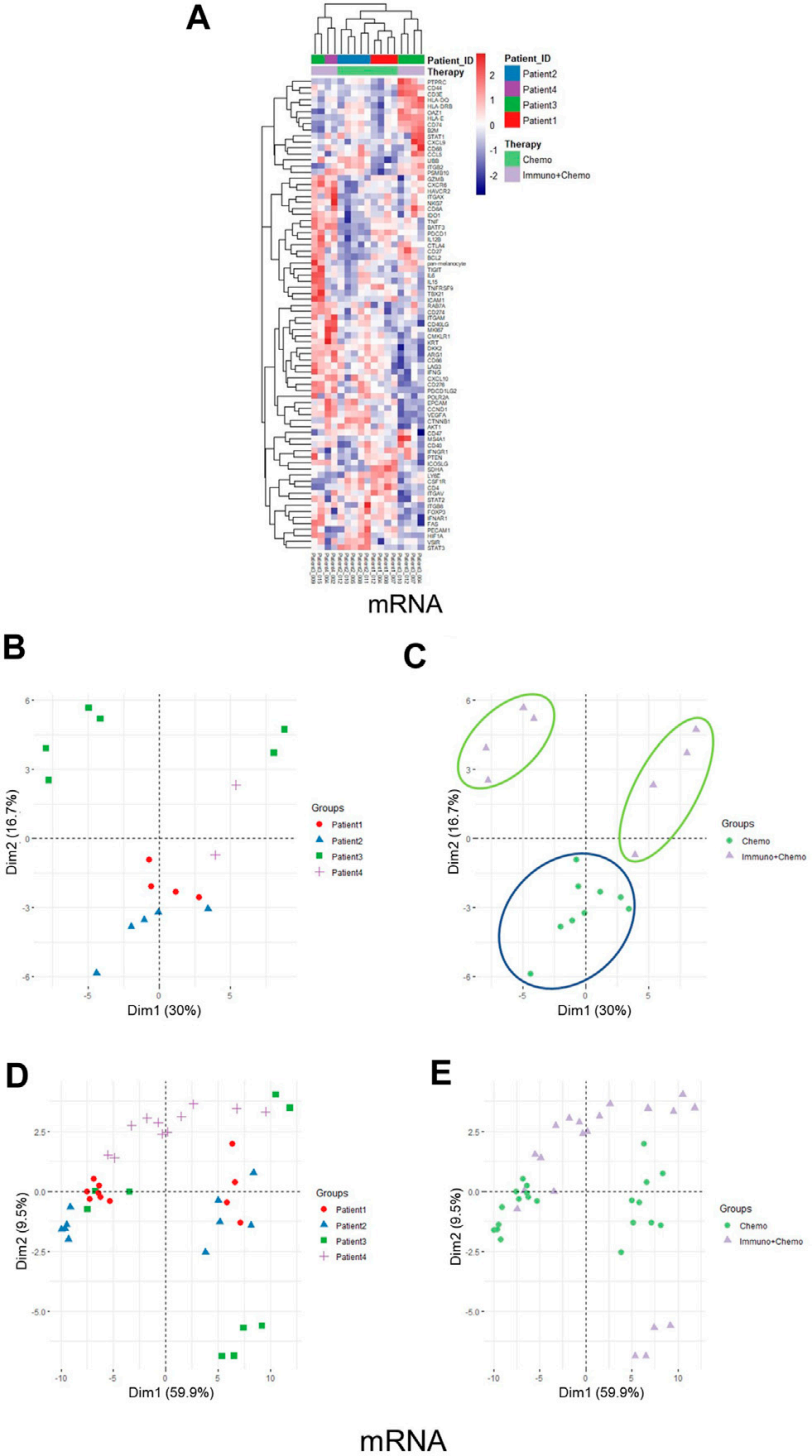
Unsupervised hierarchical clustering and PCA of stromal regions based on mRNA expression and all ROI clustering. **(A)** Unsupervised hierarchical clustering of mRNA expression in the stroma ROIs of chemotherapy or combinational therapy patients. ROIs are grouped based on treatment options and patient IDs. **(B,C)** PCA plots of mRNA expression in the stroma ROIs grouped by patients **(B)** or therapies **(C)**, respectively. **(D,E)** PCA plots of mRNA expression in all ROIs (tumor and stroma) grouped by patients **(D)** or therapies **(E)**, respectively.

Of further importance, a holistic view of the tumor region–based clustering (both unsupervised hierarchical clustering and principal component analysis (PCA)) resulted in concordant findings that the RNA expression profiling correlates strongly with therapeutic options (Figures 5A–C). Further to these findings, both unsupervised hierarchical clustering and PCA analyses also yielded two distinct therapy-guided groups at the mRNA level in the stromal regions (Figures 6A–C). These ROI region–defined grouping could not be observed when tumor and stroma are mixed (Figures 6D,E), emphasizing the importance of spatially oriented information for patient stratification and biomarker discovery.

### DSP Profiling of RNAs and Proteins Reveals Therapy-Oriented Differences

**FIGURE 7:**
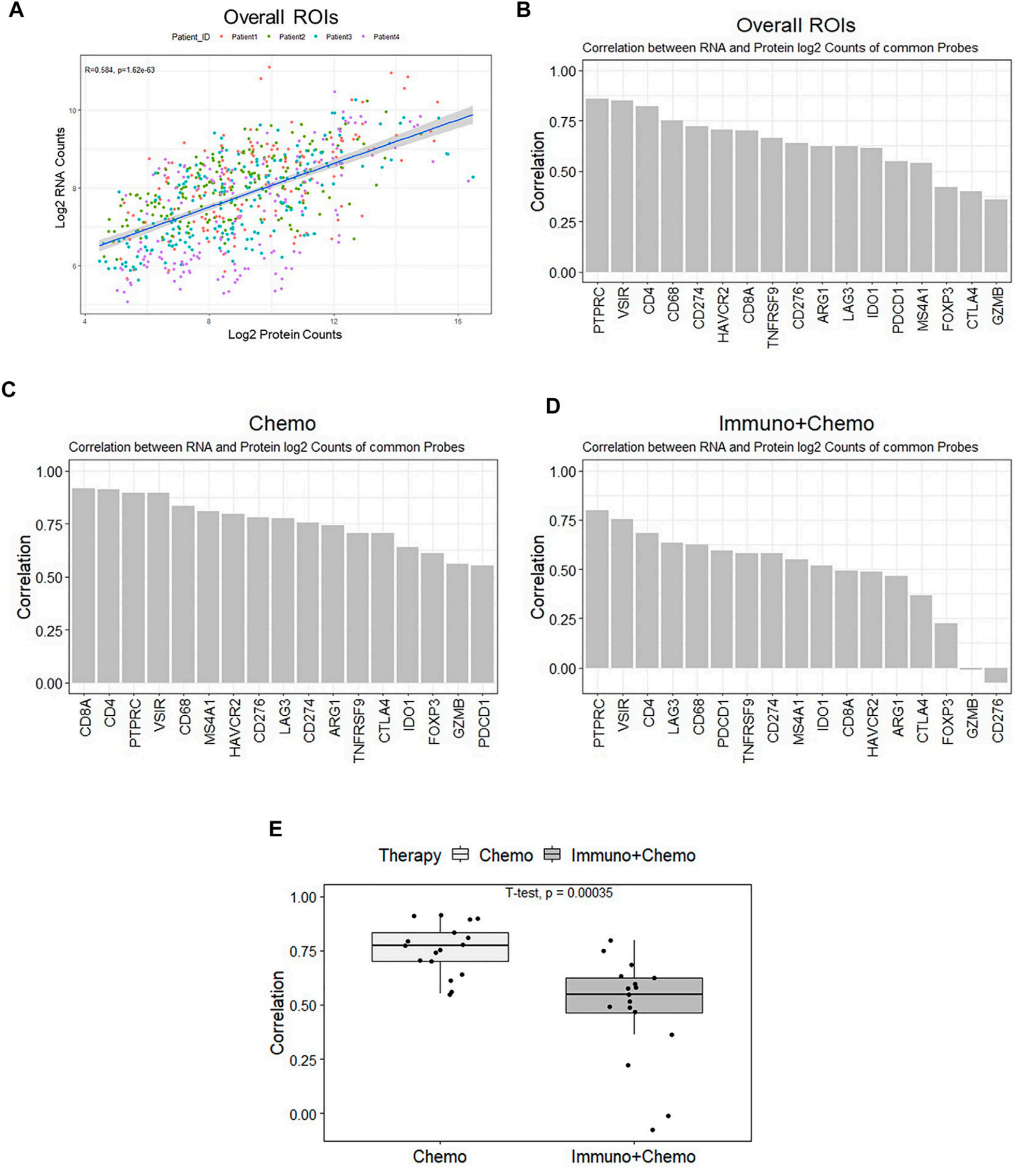
All ROI-based correlation analyses of the 17 common mRNA and protein. **(A)** Protein (x-axis) and mRNA (y-axis) expression counts were log-transformed and plotted. The correlation coefficient is 0.57 (*p* < 0.05), and colors denote individual patients. **(B)** Ranked bar chart of common protein and mRNA correlation of individual targets based on all ROIs. **(C,D)** Ranked bar charts of common protein and mRNA correlation of individual targets for chemo and chemo/immune patient groups. **(E)** Boxplot of individual ROI correlation based on common genes between therapies.

We compared the DSP profiling of RNAs and proteins in common within the same regions at the subhistological resolution of a few hundred square micrometers in size. The purpose was to gain insights into the dynamic changes between RNA and protein. Seventeen common targets [AGR1, CD4, CD8 (CD8A), and CD20 (MS4A1), CD68, CTLA4 (cytotoxic T-lymphocyte–associated protein 4), GZMB, 4-1BB (TNFRSF9), IDO1, LAG3, PD-1 (PDCD4), CD45 (PTPRC), PDL1 (CD274), FOXP3 (forkhead box P3), VISTA (V-domain immunoglobulin suppressor of T cell activation), B7-H3 (CD276), and Tim-3 (T-cell immunoglobulin and mucin domain 3)] were filtered out and plotted based on their expression correlation for individual ROIs (Figure 7A). An overall correlation between RNAs and proteins was moderate (*R* ^2^ = 0.58, *p* < 0.05). Genes including PD-L1, CD4, and VISTA exhibited strong correlations at the RNA and protein levels. CTLA4, FOXP3, and GZMB exhibited weak correlations (Figure 7B). The transcriptomic and proteomic correlation differed between therapy groups, with an overall higher correlation in the chemotherapy group than in the combinational therapy group (*p* < 0.05) (Figures 7C–E). The most pronounced alteration of GZMB and CD276 showed high concordance in the chemotherapy patients but minimal or negative correlations in combination therapy patients (Figures 7C,D). Despite the likelihood of low levels of transcripts in specific ROIs (in the case of *GZMB* and *CD276*), low correlations of FOXP3 and CTLA4 due to the low level of protein below the baseline suggest that transcriptional information may not be fully interpreted at protein levels to execute their biological function. Therefore, posttranslational modifications likely play pivotal roles in a particular cellular context (Shiratori et al., 1997; Chikuma et al., 2000).

## Discussion

High-plex profiling, specially combining proteomic and transcriptomic data on the same tissue section at the sub-histological levels, is challenging to be achieved. Previously, the TMEs of non–small-cell lung cancer (NSCLC), melanoma, pancreatic cancer, prostate cancer, bladder cancer, soft tissue sarcoma, and breast cancer were analyzed using DSP, but mainly on the protein expression (Ihle et al., 2019; Toki et al., 2019; Cabrita et al., 2020; Gundle et al., 2020; Stewart et al., 2020; Zugazagoitia et al., 2020). This is likely due to either their protein-focused study design or the archived samples in the studies such as the TMAs are practically difficult for the quantitative RNA profiling.

This study looked at the spatial-directed proteogenomic profiling on 4 Stage III CRC formalin-fixed paraffin-embedded (FFPE) patient samples to provide insights into the therapeutic mechanisms and biomarkers discovery, and compare the biological differences between mRNA and protein under the same experimental setting. The correlation matrix provided a distinct association between ROIs and genes, respectively, with strong tumoral and stromal ROI clusters confirming the robustness of the technology. The immune and epithelial markers at mRNA and protein levels were also co-clustered separately, suggesting a strong association of those genes within the particular TMEs. Our results are in line with the other single-cell analyses of CRC patients, where they showed co-clustering of different T-cell subpopulations (Zhang et al., 2018; Lee et al., 2020). Comparison between therapy-guided expression at both the mRNA and protein levels showed high consensus between the common biomarkers to differentiate tumor from stroma regions (higher level of EpCAM, pan-cytokeratin, and ki67), indicating general robustness of this technology for proteogenomic co-profiling. The only difference of elevated *VEGFA* (vascular endothelial growth factor A) at the mRNA level associated with chemotherapy alone may be caused by the protein panels non-inclusive of that target (Supplementary Figures S4A–F). The stromal markers expressed more dynamic alterations upon therapeutic induction, and common pathways altered in the stroma in response to both therapies mainly include cytokine regulation, T-cell activation and selection, cell adhesion molecule regulation, interleukin-2 production, myeloid leukocyte activation, and antigen processing and presentation of both at the RNA and protein levels (Supplementary Figures S5A–C and Figures 6A–C).

From a refined TME perspective, we were able to distinguish the tumor and stroma region–associated specific expression programs with the discovery of PD-L1 high expression at both the mRNA and protein levels in combinational therapy–treated CRC patients and confirmed with follow-up IHC staining. Considering all four patients are MSS based on the IHC data (nuclear expression of MLH1 (MutL homolog 1), MSH2 (MutS homolog 2), MSH6 (MutS homolog 6), and PMS2 (PMS1 homolog 2, mismatch repair system component)) (Supplementary Figure S3), a high level of PD-L1 (CD274) within specific TMEs may evoke a better immunogenic response and serve as a prognostic marker for immunotherapies for MSS CRC patients. A subset of immune markers was concomitantly elevated in the combination therapy group (chemo + immuno), suggesting more dynamic immunoactivities in tumor-associated regions therein. In particular, elevated *B7-H3* mRNA is largely in line with a recent large cohort IHC profiling showing an increasing pattern of this molecule in primary CRC and correlated partially with the PD-L1 expression. Our finding of high expression of IDO1 at the proteogenomic levels coincides with TCGA (The Cancer Genome Atlas) and other studies, where higher expression of *IDO1* at the mRNA level was observed in primary CRC (Guil-Luna et al., 2020; Kitsou et al., 2020; Le Naour et al., 2020). Given the limitation of bulk-tissue analysis for biomarker discovery, our finding further strengthened the bona fide importance of assessing the IDO1 expression pattern in TMEs, consequently providing helpful information for clinical study design for checkpoint inhibitors. Increased common immune marker CD45 at the protein level dictates the importance of extending discovery from the transcriptional level to the proteomic level and may suggest proteins are likely to serve as better biomarkers than mRNA in the TME under a specific context. Indeed, many immune-related mRNA expressions failed to be prognostic factors in colorectal carcinoma (Lu et al., 2020).

Evidence is furthered by looking at the tumor surrounding stroma where upregulation of STING and CD14 was observed in chemotherapy-only patients, suggesting a lack of positive immune infiltration in these patients. This finding is also consistent with the recent discovery of STING as an independent prognostic factor in the early- and late-stage CRC, indicating a critical role of STING in regulating immunogenic response (Chon et al., 2019). Considering STING agonists are being developed in clinical trials, this may infer a newly explorable direction for combinational clinical regimes (Chon et al., 2019; Pan et al., 2020). A study of 298 CRC patients also found that the CD14 level was lower in tumors than adjacent normal tissues, indicating a potential role of monocytes/macrophages in driving checkpoint inhibitor–mediated antitumor effect. Tumor region–defined compartmentalization also revealed therapy-specific expression signatures at the mRNA level, and these distinct features were evident at both the tumor and stroma regions. This expression profiling–based patient stratification cannot be identified when the tumor and stroma data are mixed at the bulk level, again implying the significance of interrogating spatial information and sample purity for mechanism elucidation and biomarker discovery. Although statistical power could be reinforced by analyzing multiple ROIs with similar expression characteristics, considering only two patients in each group, many of these findings must be further validated with more patient samples. Nevertheless, with the precision of the ROI selection technique, we were unprecedently capable of comparing the protein and RNA expression of specific important markers in the TMEs, which has not been conducted in CRC in such a fine-toned manner. Although general proteogenomic correlation is decent, different genes show various degrees of expression consistency. The low consistency of key molecules CTLA4 and FOXP3 is due to the low expression at the protein level. Posttranslational modifications of those two proteins were discovered, providing additional insights into the TME (Shiratori et al., 1997). Moreover, the overall correlation is lower in combinational therapy–treated patients, indicating a more dynamic proteogenomic interplay in specific patients, likely due to either different tumor biology intrinsically or treatment-associated causations.

Despite the findings we made here, our study is limited in part by patient numbers. Although the ROI-based individual datasets have proven to be reliable both technically and biologically, statistic stringency and the power of the analysis have to be further strengthened by exploring additional CRC patients. Paired pretreatment biopsy samples may reveal more information to identify how tumors evolve and adapt in response to chemotherapy with or without coadministration of checkpoint blockades and may help design future clinical strategies in CRC. Nevertheless, by employing DSP, with only limited samples, high-plex proteogenomic profiling of over 40-plex proteins and 84-plex mRNA on CRC patients was depicted at a very high resolution, which has never been achieved with previous methods (Wang et al., 2021b). In summary, we provided valuable research resources and preliminary data by focusing on specific TME regions histologically unobtainable with other technologies and ensured future application in various contexts to disentangle underlying biology within the complex and heterogenous TMEs.

## Materials and Methods

### Clinical Patient Characteristics

Tumor specimens from four patients were obtained during surgery from Beijing Cancer Hospital. Samples were immediately fixed and paraffin-embedded. All patients were TNM classified as stage III colorectal cancer (T3) with no metastasis at diagnosis. Before surgery, two patients received neoadjuvant chemotherapy with capecitabine and oxaliplatin (CAPEOX). The other two patients received the CAPEOX chemotherapy combined with sintilimab (a monoclonal antibody that binds to *PD-1*). All four patients were pathologically evaluated pre-and posttreatment. All patients were microsatellite stable (MSS) based on IHC data of MLH1, MSH2, MSH6, and PMS2. Anti-MLH1 antibody (GM002), anti-MSH2 antibody (RED2), anti-MSH6 antibody (EP49), and anti-PMS2 antibody (EP51) were obtained from Gene Tech Biotechnology (Shanghai China). All four patients had histological tumor regression with AJCC/NCCN grade (TRG) of 2/3 based on posttreatment pathological evaluation. Three serial sections of tissue samples (5 μm thickness) from individuals were freshly prepared for the DSP protein, RNA profiling, and IHC, respectively, to allow parallel comparison. Detailed patient information is provided in supplementary (Supplementary Table S1). All tissue samples had informed consent from the patients as documents in Beijing Cancer Hospital for research purposes.

### Exploring Colorectal Cancer TME Using DSP at a Proteogenomic Scale

Digital spatial profiling (DSP) was performed on slide-mounted FFPE samples as described previously (Wang et al., 2021a). For protein profiling, slides were preprocessed with deparaffinization and rehydration before incubation with a tricolor fluorescence morphology marker panel targeting Pan-CK (epithelial and tumoral regions), CD45 (immne cells), and SYTO13 (nuclear) together with target-specific oligo-conjugated primary antibodies cocktails for 40 proteins. For RNA profiling, a panel of probes for 84 mRNAs were hybridized at 37°C overnight in a hybridization oven and then incubated with the aforementioned morphological antibody panel. Detailed protein and RNA target information can be found in supplementary (Supplementary Tables S2, S3). For both the protein and RNA profiling, multicolored morphology markers allow compartment visualization to guide the selection of regions of interest (ROIs). This one-step overnight incubation was then followed by 20x high-precision scanning on a GeoMx DSP system and circular ROI selection. To ensure reliable quantification and inter-ROI data comparison, surface areas of ROIs were drawn between 2 × 10^4^ and 1.2 × 10^5^ μm^2^ for protein profiling or between 3.8 × 10^4^ and 3.8 × 10^5^ μm^2^ for RNA profiling. Conjugated target-specific oligos were released upon UV light illumination and collected in 96-well plates. Heat-dried oligos were then hybridized to unique NanoString barcodes, purified on the nCounter Prep station, and counted on the nCounter analysis system using standard procedures (Merritt et al., 2020; Wang et al., 2021b).

### Immunohistochemistry

Immunohistochemistry (IHC) was performed on (FFPE) patient sections using prevalidated *PD-L1* antibody (13684S, Cell Signaling Technology Danvers, MA). After deparaffinization and rehydration in xylene and ethanol, antigen retrieval was performed in 1x EDTA retrieval solution (pH 9.0) with heating. Slides were then blocked with goat serum (x0907, Dako) for 1 h and followed by primary antibody incubation at 1:200 dilution. After overnight incubation, slides were incubated with goat anti-rabbit antibody (E046201, Dako), then developed with DAB substrate (k3468, Dako), and counterstained with hematoxylin (CTS-1090, Biotechnologies). Slides were scanned with a brightfield microscope (Aperio CS2, Leica) and processed by ImageScope software (Leica).

### Data Processing and Statistical Analysis

To adjust system and experimental bias and to counteract ROI size variation effects, raw digital count files (RCC) for individual ROIs were normalized by ERCC RNA spike-in controls before downstream processing. This quality control step generated normalization positive factors from individual ROIs. The ROI inclusion criteria were limited on a minimum surface area of 1.6 × 10^3^ μm^2^ for protein and 1.6 × 10^4^ μm^2^ for RNA, and minimum nuclei counts of 20 for protein and 200 for RNA generally. Any ROIs resulting in a normalization positive factor higher than 3 or lower than 0.3 were excluded from the downstream analysis. QC-qualified ROI count files were then normalized by the geometric mean of housekeeping genes (Histone H6, GAPDH, and S6 were used for protein. *RAB7A*, *OAZ1*, *UBB*, *POLR2A,* and *SDHA* were used for RNA). The normalized data were log-transformed with or without being median-centered before comparison and plotting. For proteogenomic comparison, gene and protein ID were matched by Entrez ID for the following genes: AGR1, CD4, CD8 (CD8A), CD20 (MS4A1), CD68, CTLA4, GZMB, 4-1BB (TNFRSF9), IDO1, LAG3, PD-1 (PDCD4), CD45 (PTPRC), PDL1 (CD274), FOXP3, VISTA (VSIR), B7-H3 (CD276), and Tim-3 (HAVCR2). All data were processed and analyzed in DSP analysis software and R version 3.6.0 with relevant packages. The correlation analysis was computed using the “Pearson” method. Hierarchical clustering and correlation matrix were done with “pheatmap” package. The principal component analysis (PCA) was conducted by “FactoMineR” and “factoextra” packages. Volcano plots were created with log2FC set at 1 and adjusted *p*-value at 0.05 for cut-off (dashed lines). Venn plots were created with the “VennDiagram” package. For the differential expression analysis, a non-parametric Mann–Whitney U-test was used, and *p*-value was set to 0.05 at a significant cut-off. Due to the limited number of probes and samples, in some cases, *p*-value was presented without adjustment. Other relevant plots were generated by “ggplot2” package. For function and pathway annotation and enrichment analysis, differentially expressed genes (gene symbol) were submitted and processed in Metascape online interface (https://metascape.org).

## Data Availability

The raw data supporting the conclusions of this article will be made available by the authors, without undue reservation, to any qualified researcher.

## Supplementary Material

Supplementary Figure 1 Full section images and ROI selection strategies for additional patients. The multicolor labeled whole tissue sections are shown on the left panels (20x magnification), and all tumor and stroma ROIs are displayed on the right panels (green: Pan-cytokeratin, red: CD45, and blue: SYTO13). **(A,B)** Chemotherapy-treated patients. **(C,D)** Chemotherapy- and immunotherapy-treated patients.

Supplementary Figure 2 Correlation matrices for protein and mRNA, respectively. **(A,B)** Gene-wise association plots based on all ROIs. 84-plex mRNA is shown on the left panel and 40-plex protein on the right. **(C,D)** ROI-wise association plots based on 17 common target expression profiles. mRNA is shown on the left and protein on the right. Color bars are grouped based on treatment options, ROI categories (tumor/stroma), and patient IDs.

Supplementary Figure 3 IHC staining of four patients. All markers are presented with representative images (100x), including MSH6, PMS2, MLH1, and MSH2, and all proteins show positive nuclear staining based on pathological assessment.

Supplementary Figure 4 Differential expression profiling of tumor *versus* stroma ROIs in two treatment groups. **(A–C)** mRNA expression profiling between tumor and stroma regions. Volcano plots show differentially expressed genes in two treatment groups, respectively (dashed lines represent log2FC=1 on the x-axis and adjusted *p*=0.05 on the y-axis). The Venn diagram shows the common and unique differentially expressed mRNA markers between therapies. **(D–F)** The protein expression profiling between tumor and stroma regions. Volcano plots show differentially expressed genes in two treatment groups, respectively (dashed lines represent log2FC=1 on the x-axis and adjusted *p*=0.05 on the y-axis). The Venn diagram shows the common and unique differentially expressed protein markers between therapies.

Supplementary Figure 5 Gene and pathway annotation of unique differentially expressed mRNA in chemotherapy groups. **(A)** The unique gene list (gene symbols) was processed and analyzed using a Metascape online tool, and functional annotations are ranked by −log10 *p*-value. **(B,C)** Enrichment network charts from unique gene lists where nodes are individual genes and color codes are gene identities or *p*-values.

Supplementary Figure 6 Gene and pathway annotation of unique differentially expressed protein in chemotherapy groups. **(A)** The unique gene list (gene symbols) was processed and analyzed using a Metascape online tool, and functional annotations are ranked by −log10 *p*-value. **(B,C)** Enrichment network charts from unique gene lists where nodes are individual genes and color codes are gene identities or *p*-values.

Supplementary Table 1 Patient clinical information.

Supplementary Table 2 DSP probe list for mRNA.

Supplementary Table 3 DSP probe list for proteins.

Supplementary Table 4 Differential expression of mRNA in tumor ROIs for two chemotherapy-treated patients. Genes are ranked by *p*-value.

Supplementary Table 5 Differential expression of mRNA in stroma ROIs for two chemotherapy-treated patients. Genes are ranked by *p*-value.
