# Supplementary material for: Tumor Microenvironment Profiles Reveal Distinct Therapy-Oriented Proteogenomic Characteristics in Colorectal Cancer
Source: Front Bioeng Biotechnol. 2021 Oct 28;9:757378. doi: 10.3389/fbioe.2021.757378 (PMC8581216; doi:10.3389/fbioe.2021.757378)
Supplement: Supplementary file 1 [file DataSheet1.PDF]

A

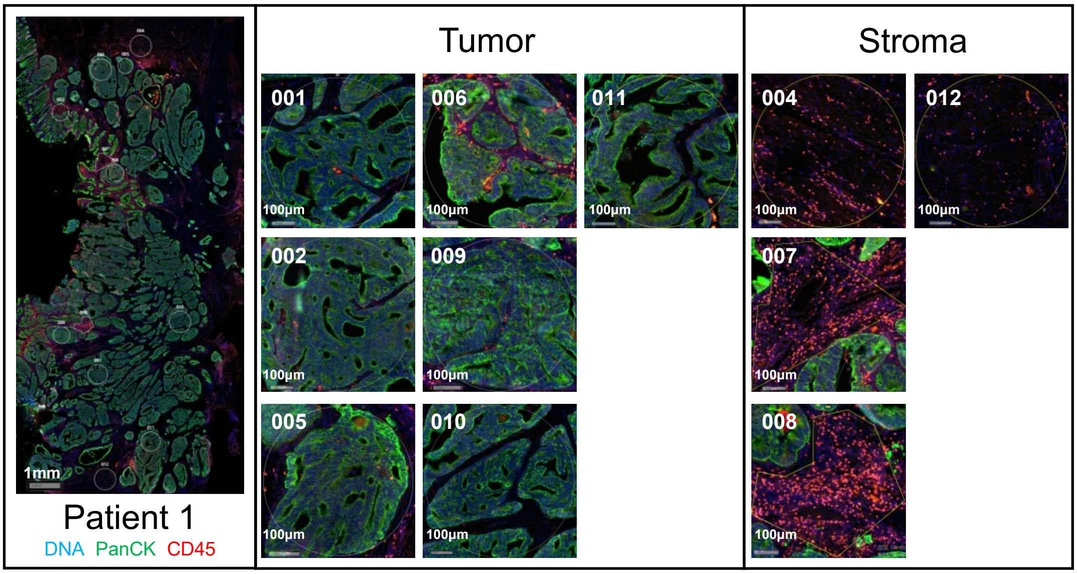

B

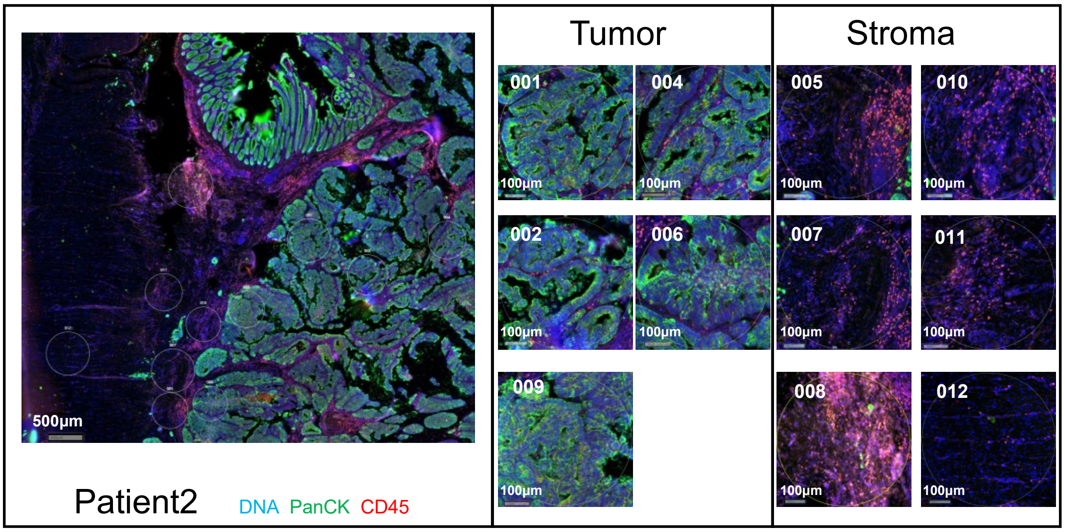

Supplementary figure 1

C

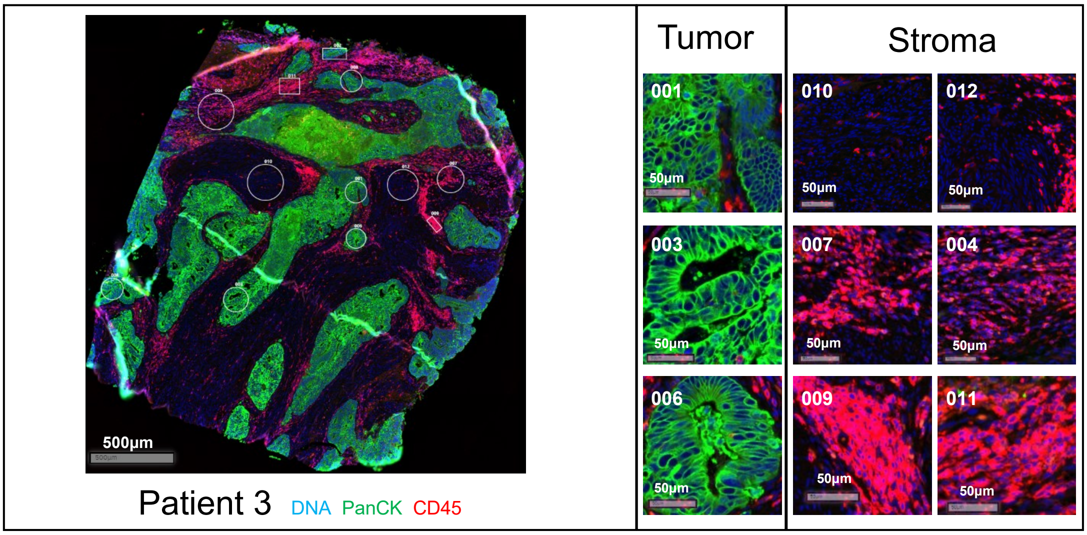

D

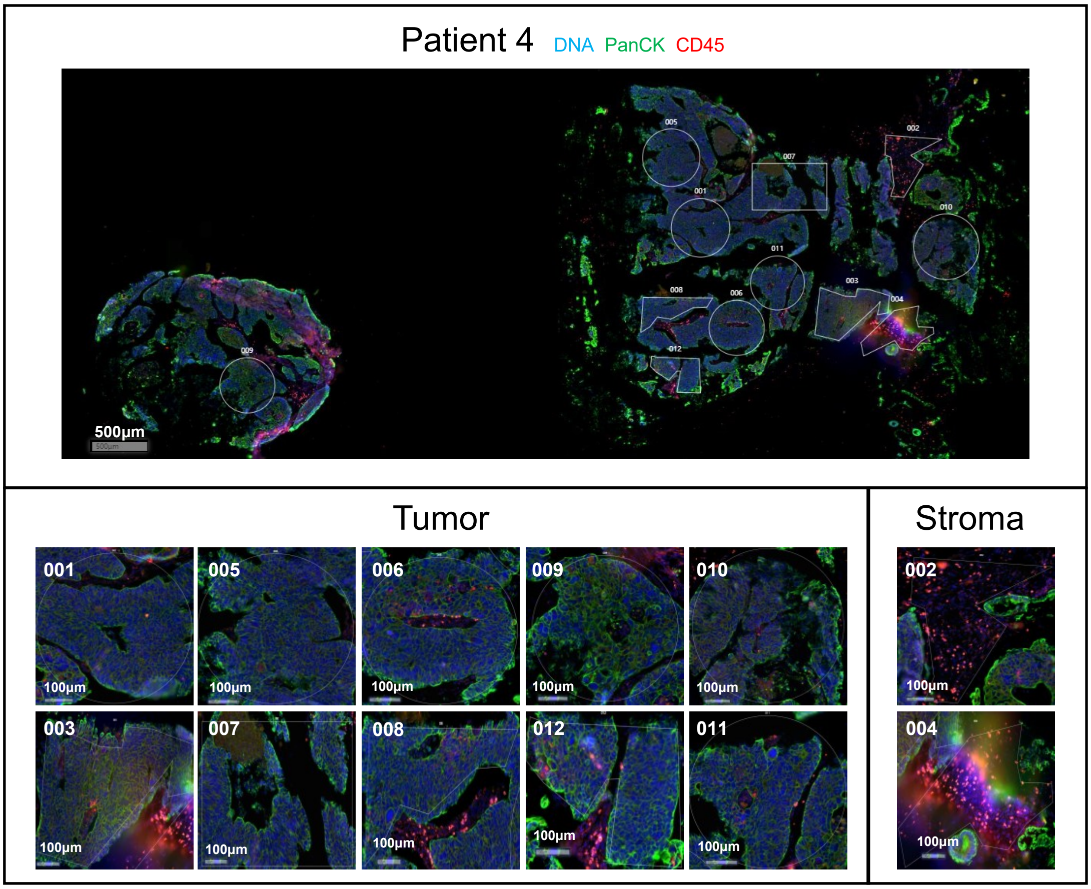

Supplementary figure 1

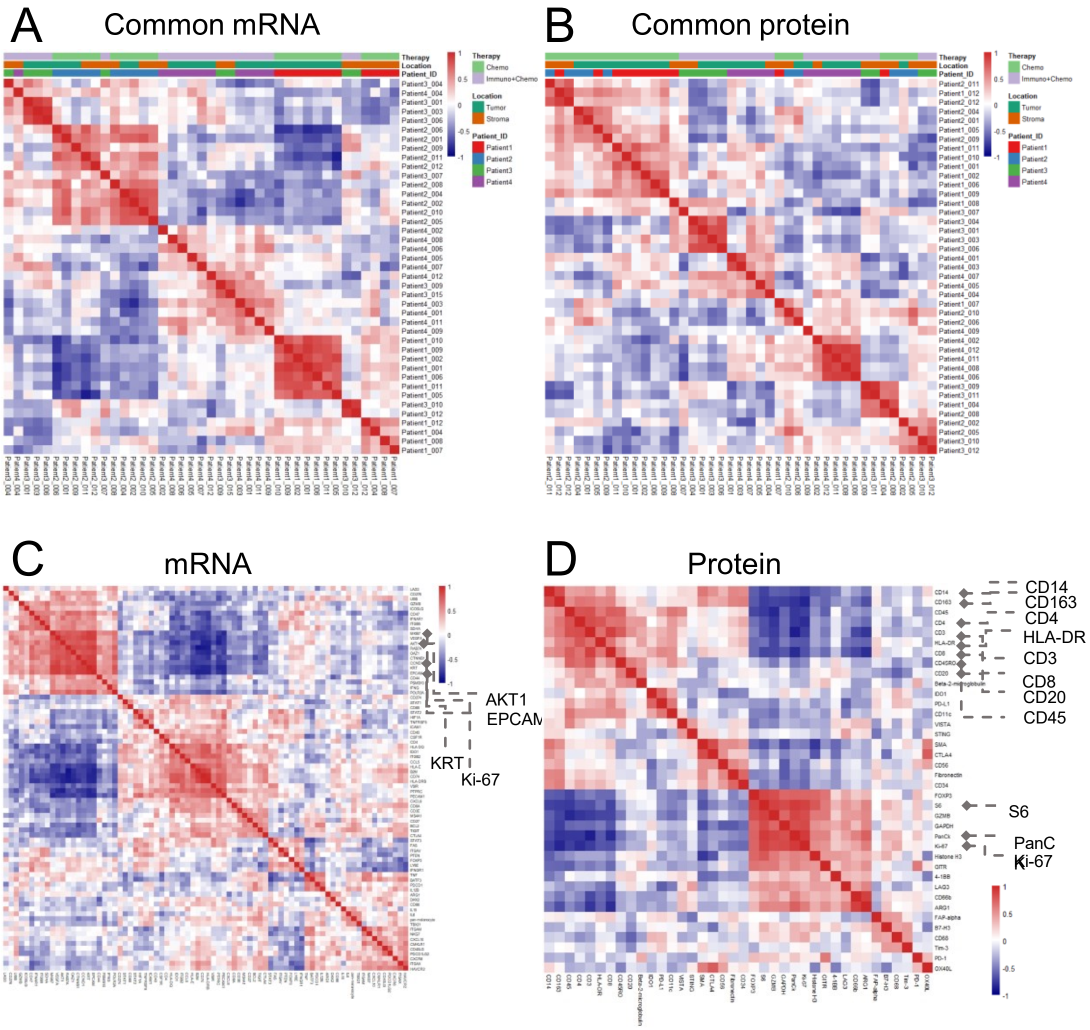

Supplementary figure 2

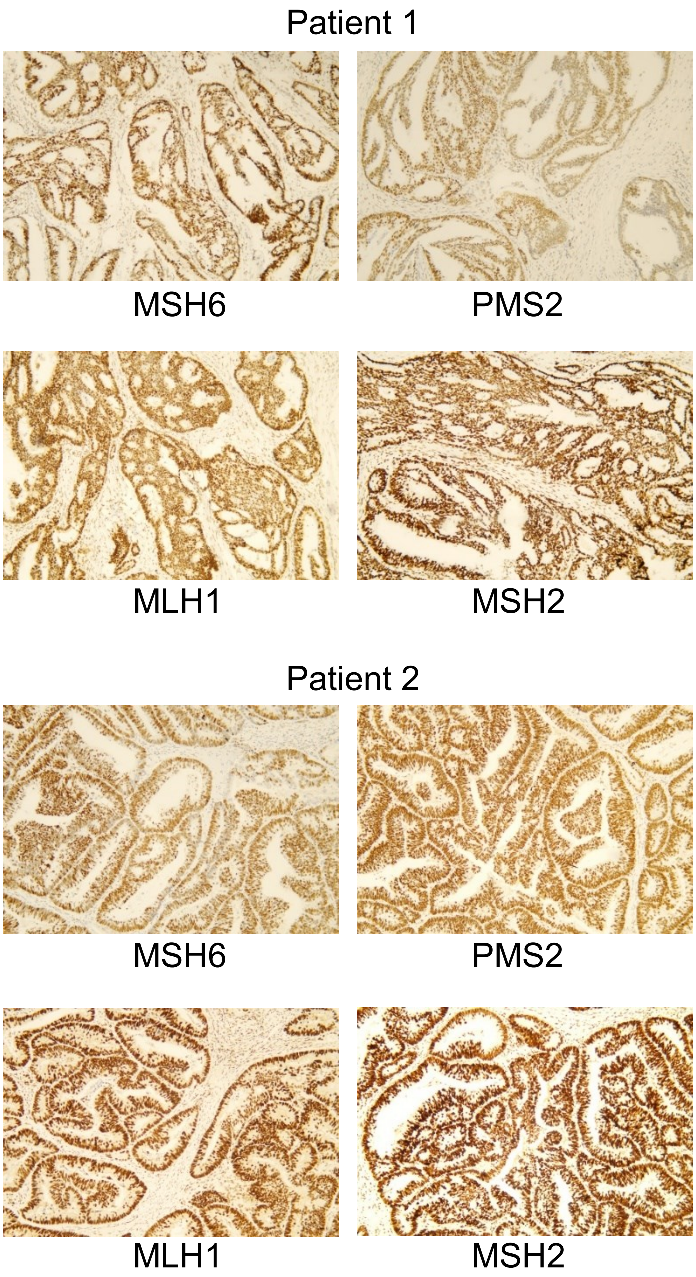

Supplementary figure 3

Patient 3

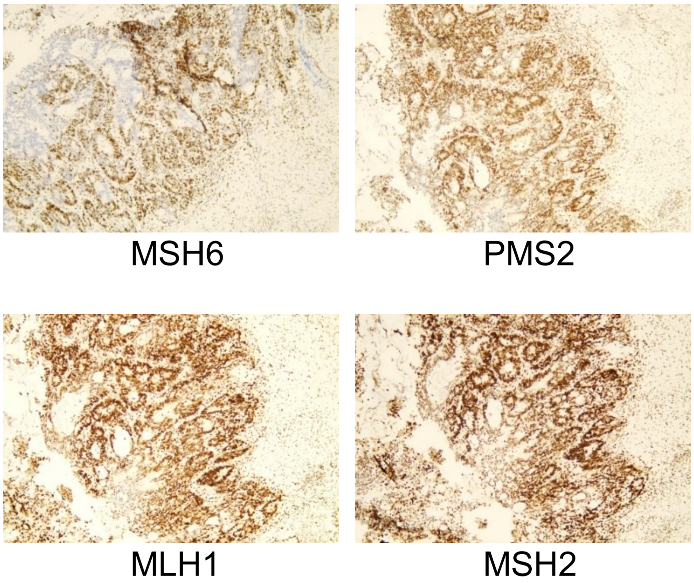

Patient 4

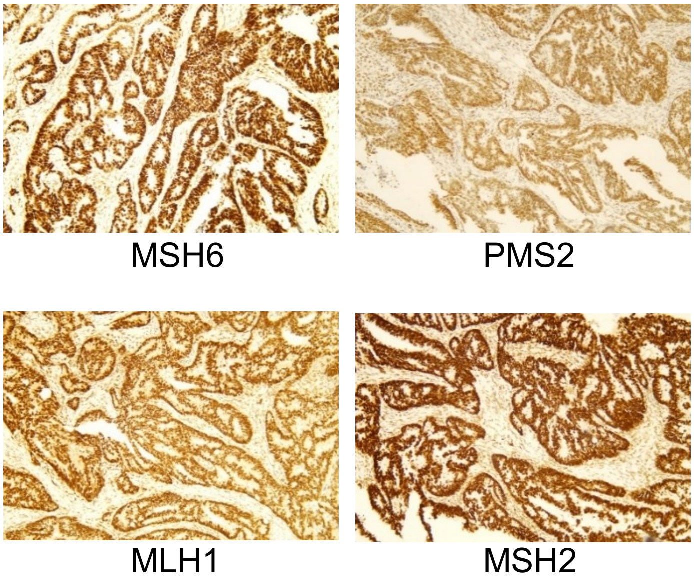

Supplementary figure 3

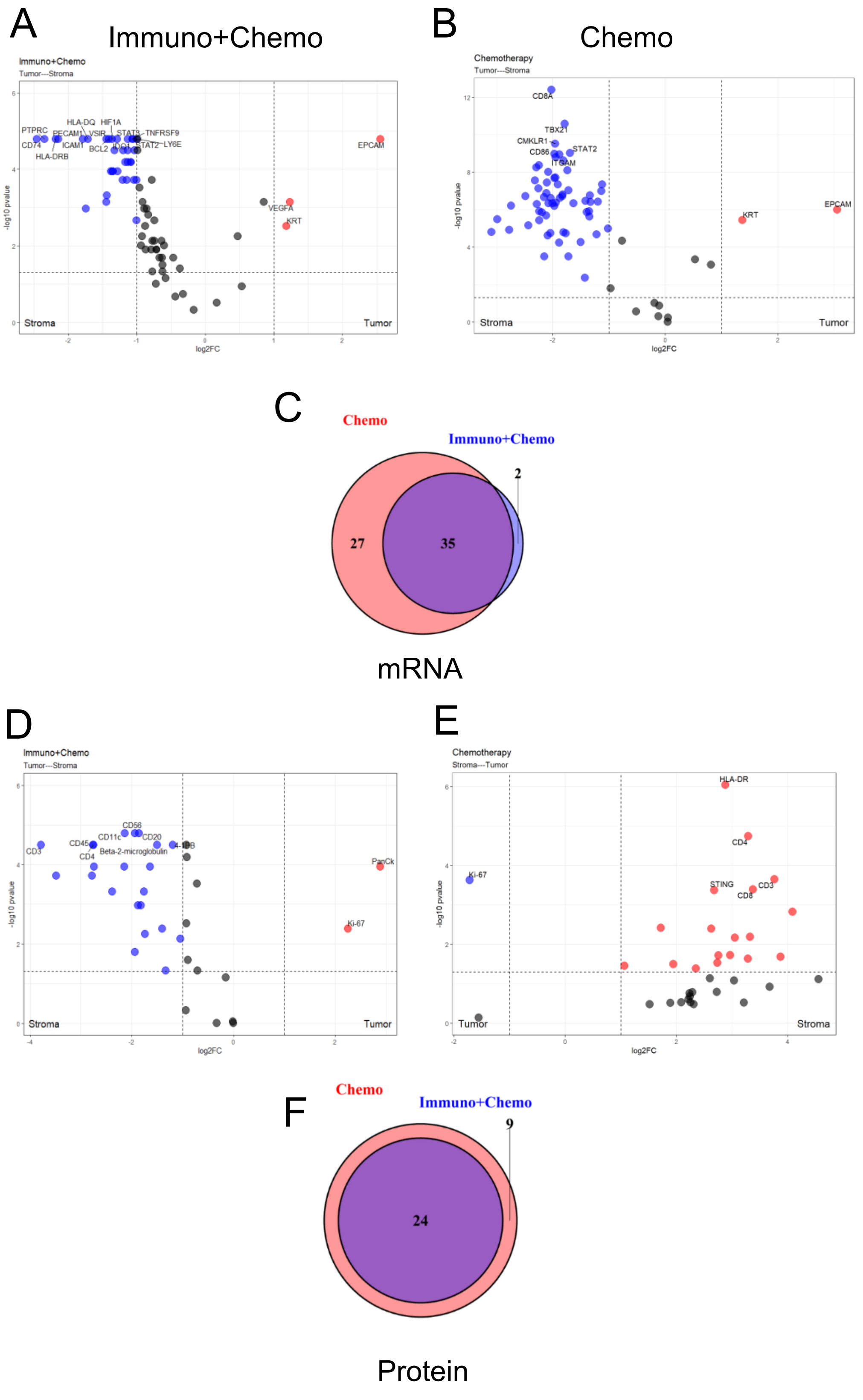

Supplementary figure 4

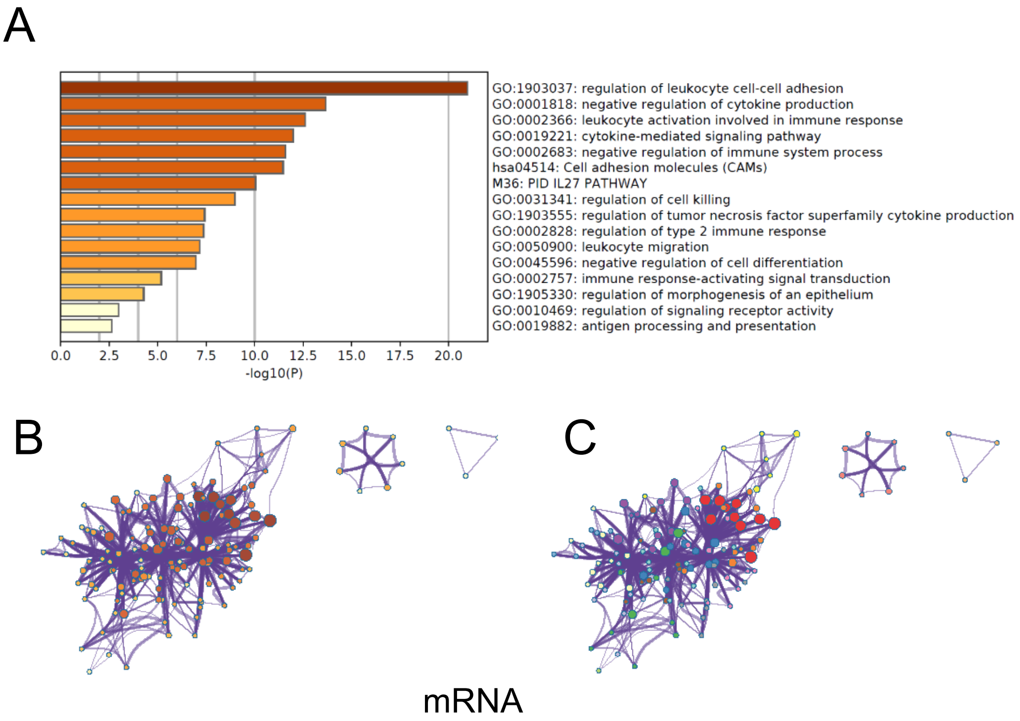

Supplementary figure 5

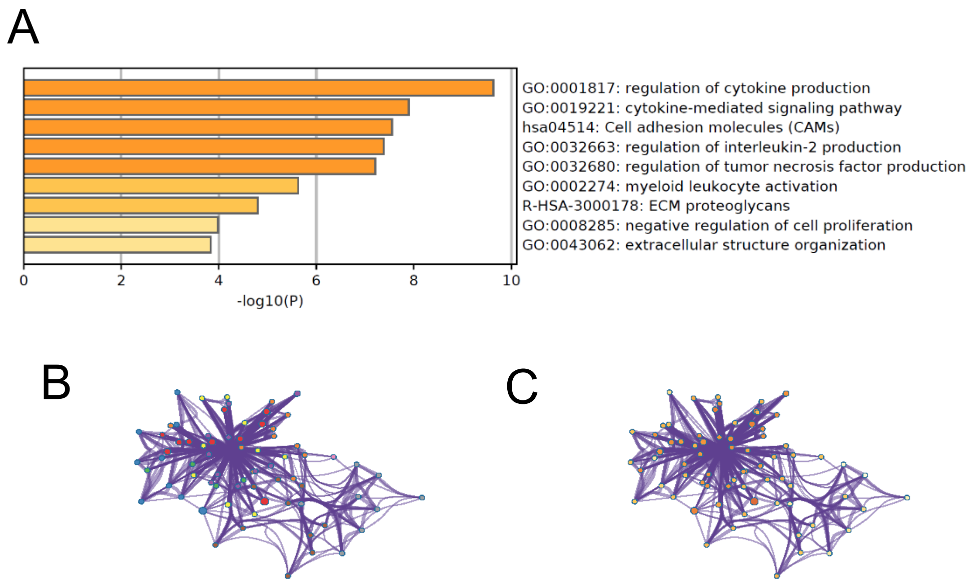

Supplementary figure 6

Patients clinical information

PatientID	Gender	Age	Before treatment		Neoadjuvant therapy	After treatment	
			Pathological type	cTNM classification		IHC results	pTNM classification
Patient1	Male	67	Rectal moderately differentiated adenocarcinoma	T4aN2b	CAPEOX	Cmet(2+),EGFR(2+),HER2(1+),KI67(+80%),MLH1(+),MSH2(+),MSH6(+),PMS2(+)	ypT3N1a
Patient2	Female	69	Rectal moderately differentiated adenocarcinoma		CAPEOX	Cmet(2+),EGFR(2+),HER2(1+),KI67(>75%),MLH1(+),MSH2(+),MSH6(+),PMS2(+)	ypT3N1b
Patient3	Female	66	Rectal moderately differentiated adenocarcinoma	cT3cN2	CAPEOX+Sintilimab	Cmet(2+),EGFR(2+),HER2(0),KI67(90%+),MLH1(+),MSH2(+),MSH6(+),PMS2(+)	ypT3N0
Patient4	Male	49	Rectal poorly differentiated adenocarcinoma		CAPEOX+Sintilimab	BRAF(-),Cmet(-),EGFR(2+),HER2(3+),KI67(>75%),MLH1(+),MSH2(+),MSH6(+),PMS2(+)	ypT3N1a

Supplementary Table 1

mRNA Panel

Panel	Probe					
GeoMx^{TM} RNA Panel for Immuno-Oncology	CCL5	LAG3	CD3E	CXCL10	IFNGR1	Multi-CK
	CD27	NKG7	CD4	DKK2	IL12B	pan-Melanoma
	CD274 (PDL1)	PSMB10	CD40	EPCAM	IL15	PDCD1
	CD276 (B7-H3)	PDCD1LG2	CD40LG	FAS	IL6	PECAM1
	CD8A	STAT1	CD44	FOXP3	ITGAM (CD11B)	PTEN
	CMKLR1	TIGIT	CD47	GZMB	ITGAV	PTPRC (CD45)
	CXCL9	AKT1	CD68	HAVCR2 (TIM3)	ITGAX (CD11C)	STAT2
	CXCR6	ARG1	CD74	HIF1A	ITGB2	STAT3
	HLA-DQA1/2	B2M	CD86	ICAM1	ITGB8	TBX21
	HLA-DRB	BATF3	CSF1R	ICOSLG	LY6E	TNF
	HLA-E	BCL2	CTLA4	IFNAR1	MKI67	TNFRSF9 (41-BB)
	IDO1	CCND1	CTNNB1	IFNG	MS4A1 (CD20)	VEGFA
	VSIR (VISTA)	OAZ1	POLR2A	RAB7A	SDHA	UBB
	Neg Probe 1	Neg Probe 2	Neg Probe 3	Neg Probe 4	Neg Probe 5	Neg Probe 6

Supplementary Table 2

Protein Panel

Panel	Probe					
	Beta-2-microglobulin	CD3	CD56	CTLA4	GZMB	PD-1
Immune Cell Profiling Panel	CD11c	CD4	CD68	Pan-cytokeratin	HLA-DR	PD-L1
Human Protein Core	CD20	CD45	CD8	Fibronectin	MS IgG2a	MS IgG1
	Ki67	SMA	S6	GAPDH	Histone H3	Rb IgG
IO Drug Target Panel Human	4-1BB	LAG3	ARG1	OX40L	GITR	TIM-3
Protein Module	B7-H3	STING	IDO1	VISTA		
Immune Cell Typing Panel	CD14	FAPalpha	CD163	FOXP3	CD34	CD45RO
Human Protein Module	CD66b					

Supplementary Table 3

Differential expression table of Patient 1 vs 2 (Therapy: Chemo) in Tumor ROIs

Gene	Log2FC	Pvalue	Adjusted p-value	-log10 p-value	-log10 adjusted p-value
CD8A	0.70438278	0.002525253	0.004726755	2.5976952	2.3254369
pan-melanocyte	0.79352126	0.002525253	0.004726755	2.5976952	2.3254369
IL6	1.3031856	0.002525253	0.004726755	2.5976952	2.3254369
DKK2	0.5059748	0.002525253	0.004726755	2.5976952	2.3254369
TNFRSF9	1.13294666	0.002525253	0.004726755	2.5976952	2.3254369
TIGIT	0.71023667	0.002525253	0.004726755	2.5976952	2.3254369
PDCD1	1.84552138	0.002525253	0.004726755	2.5976952	2.3254369
ITGAX	1.06127234	0.002525253	0.004726755	2.5976952	2.3254369
ITGAV	0.77264692	0.002525253	0.004726755	2.5976952	2.3254369
STAT2	0.42942532	0.002525253	0.004726755	2.5976952	2.3254369
GZMB	2.03146329	0.002525253	0.004726755	2.5976952	2.3254369
ICOSLG	1.26812826	0.002525253	0.004726755	2.5976952	2.3254369
CXCR6	0.83506916	0.002525253	0.004726755	2.5976952	2.3254369
TBX21	0.95504942	0.002525253	0.004726755	2.5976952	2.3254369
STAT3	-1.16874843	0.002525253	0.004726755	2.5976952	2.3254369
MS4A1	1.2338302	0.002525253	0.004726755	2.5976952	2.3254369
NKG7	0.95990026	0.002525253	0.004726755	2.5976952	2.3254369
CXCL10	0.9394517	0.002525253	0.004726755	2.5976952	2.3254369
TNF	1.15485697	0.002525253	0.004726755	2.5976952	2.3254369
CMKLR1	0.54638747	0.002525253	0.004726755	2.5976952	2.3254369
IFNAR1	0.91462922	0.002525253	0.004726755	2.5976952	2.3254369
CXCL9	1.1074965	0.002525253	0.004726755	2.5976952	2.3254369
KRT	-0.63101392	0.002525253	0.004726755	2.5976952	2.3254369
ITGB8	1.78575897	0.002525253	0.004726755	2.5976952	2.3254369
FAS	0.70627372	0.002525253	0.004726755	2.5976952	2.3254369
HIF1A	1.06963009	0.002525253	0.004726755	2.5976952	2.3254369
CD27	0.9304176	0.002525253	0.004726755	2.5976952	2.3254369
BATF3	1.10566833	0.002525253	0.004726755	2.5976952	2.3254369
CD40	0.97683757	0.002525253	0.004726755	2.5976952	2.3254369
BCL2	1.01808073	0.002525253	0.004726755	2.5976952	2.3254369
IL12B	1.06677	0.002525253	0.004726755	2.5976952	2.3254369
PTEN	1.30152909	0.002525253	0.004726755	2.5976952	2.3254369
CTLA4	0.66404178	0.002525253	0.004726755	2.5976952	2.3254369
EPCAM	-0.73417335	0.002525253	0.004726755	2.5976952	2.3254369
CD86	0.62008273	0.002525253	0.004726755	2.5976952	2.3254369
HAVCR2	0.8368252	0.002525253	0.004726755	2.5976952	2.3254369
CD4	0.80908099	0.002525253	0.004726755	2.5976952	2.3254369

Continued

Gene	Log2FC	Pvalue	Adjusted.pvalue	-log10 pvalue	-log10 adjusted pvalue
ICAM1	0.98621137	0.002525253	0.004726755	2.5976952	2.3254369
AKT1	0.68198868	0.002525253	0.004726755	2.5976952	2.3254369
CTNNB1	-0.77891726	0.005050505	0.008193042	2.2966652	2.0865548
ITGAM	0.78937245	0.005050505	0.008193042	2.2966652	2.0865548
FOXP3	0.57166455	0.005050505	0.008193042	2.2966652	2.0865548
CCND1	0.31649876	0.005050505	0.008193042	2.2966652	2.0865548
CD3E	0.62402204	0.005050505	0.008193042	2.2966652	2.0865548
PECAM1	0.9241604	0.005050505	0.008193042	2.2966652	2.0865548
PTPRC	0.41185347	0.01010101	0.015688803	1.9956352	1.8044102
PDCD1LG2	0.40612703	0.01010101	0.015688803	1.9956352	1.8044102
ARG1	0.56844195	0.017676768	0.026883418	1.7525971	1.5705155
CD276	0.3052021	0.047979798	0.067356255	1.3189416	1.1716221
CD40LG	0.43963129	0.047979798	0.067356255	1.3189416	1.1716221
IFNG	0.62693518	0.047979798	0.067356255	1.3189416	1.1716221
CCL5	0.34574746	0.047979798	0.067356255	1.3189416	1.1716221
CD68	0.38505696	0.073232323	0.100867162	1.1352972	0.9962502
CD44	0.28587206	0.106060606	0.133490073	0.9744459	0.874551
LY6E	0.35498548	0.106060606	0.133490073	0.9744459	0.874551
MKI67	0.27740323	0.106060606	0.133490073	0.9744459	0.874551
CSF1R	0.44352771	0.106060606	0.133490073	0.9744459	0.874551
VEGFA	0.30488197	0.106060606	0.133490073	0.9744459	0.874551
IDO1	0.21520389	0.148989899	0.184343434	0.8268432	0.7343723
LAG3	0.16003504	0.202020202	0.237862496	0.6946052	0.623674
VSIR	0.16925596	0.202020202	0.237862496	0.6946052	0.623674
ITGB2	-0.29449843	0.202020202	0.237862496	0.6946052	0.623674
HLA-E	0.14782366	0.267676768	0.300621601	0.5723893	0.5219798
CD274	0.22810873	0.267676768	0.300621601	0.5723893	0.5219798
IL15	0.19871956	0.267676768	0.300621601	0.5723893	0.5219798
HLA-DQ	0.29390389	0.343434343	0.358152958	0.4641563	0.4459315
CD74	-0.78743435	0.343434343	0.358152958	0.4641563	0.4459315
PSMB10	0.13794773	0.343434343	0.358152958	0.4641563	0.4459315
HLA-DRB	-0.51748147	0.343434343	0.358152958	0.4641563	0.4459315
B2M	-0.2510892	0.343434343	0.358152958	0.4641563	0.4459315
STAT1	0.12848307	0.431818182	0.443982074	0.3646991	0.3526346
CD47	0.0968485	0.53030303	0.53766835	0.2754759	0.2694855
IFNGR1	0.04278264	0.755050505	0.755050505	0.122024	0.122024

Supplementary Table 4

Differential expression table of Patient 1 vs 2

(Therapy: Chemo) in Stroma ROIs

Gene	Log2FC	P-value	Adjusted p-value	-log10 p-value	-log10 adjusted p-value
PDCD1	1.313104	0.015873	0.105339	1.799341	0.97741
ITGB2	-0.69375	0.015873	0.105339	1.799341	0.97741
GZMB	0.58373	0.015873	0.105339	1.799341	0.97741
STAT3	-0.55651	0.015873	0.105339	1.799341	0.97741
PSMB10	-0.50728	0.015873	0.105339	1.799341	0.97741
TNF	0.78015	0.015873	0.105339	1.799341	0.97741
CD27	0.400085	0.015873	0.105339	1.799341	0.97741
BATF3	0.520744	0.015873	0.105339	1.799341	0.97741
BCL2	0.676409	0.015873	0.105339	1.799341	0.97741
IL12B	0.671819	0.015873	0.105339	1.799341	0.97741
CCL5	-0.67401	0.015873	0.105339	1.799341	0.97741
CTNNB1	-0.66914	0.031746	0.165533	1.498311	0.781116
ICOSLG	0.414815	0.031746	0.165533	1.498311	0.781116
MKI67	-0.25928	0.031746	0.165533	1.498311	0.781116
VSIR	-0.43879	0.063492	0.257496	1.197281	0.58923
LY6E	0.316149	0.063492	0.257496	1.197281	0.58923
CD40LG	-0.36686	0.063492	0.257496	1.197281	0.58923
VEGFA	-0.44989	0.063492	0.257496	1.197281	0.58923
ITGAV	0.254196	0.111111	0.311966	0.954243	0.505893
CD3E	-0.31313	0.111111	0.311966	0.954243	0.505893
TBX21	0.193912	0.111111	0.311966	0.954243	0.505893
STAT1	-0.28392	0.111111	0.311966	0.954243	0.505893
MS4A1	0.333993	0.111111	0.311966	0.954243	0.505893
CD68	-0.33685	0.111111	0.311966	0.954243	0.505893
HIF1A	-0.54642	0.111111	0.311966	0.954243	0.505893
AKT1	-0.25306	0.111111	0.311966	0.954243	0.505893
CCND1	-0.48742	0.190476	0.375804	0.720159	0.425038
TNFRSF9	0.419479	0.190476	0.375804	0.720159	0.425038
ITGAX	0.343695	0.190476	0.375804	0.720159	0.425038
CXCR6	0.20214	0.190476	0.375804	0.720159	0.425038
NKG7	0.194891	0.190476	0.375804	0.720159	0.425038
IFNGR1	0.225614	0.190476	0.375804	0.720159	0.425038
B2M	-0.33448	0.190476	0.375804	0.720159	0.425038
FAS	-0.22232	0.190476	0.375804	0.720159	0.425038
PTEN	0.156263	0.190476	0.375804	0.720159	0.425038
CD4	0.296103	0.190476	0.375804	0.720159	0.425038
ICAM1	0.57261	0.190476	0.375804	0.720159	0.425038

Continued

Gene	Log2FC	Pvalue	Adjusted.pvalue	-log10 pvalue	-log10 adjusted pvalue
ICAM1	CD44	-0.24032	0.285714	0.496599	0.544068
AKT1	DKK2	-0.29811	0.285714	0.496599	0.544068
CTNNB1	CD74	-0.4148	0.285714	0.496599	0.544068
ITGAM	HLA-DRB	-0.52376	0.285714	0.496599	0.544068
FOXP3	CTLA4	0.22461	0.285714	0.496599	0.544068
CCND1	CD274	-0.20324	0.412698	0.641	0.384367
CD3E	CXCL10	-0.16982	0.412698	0.641	0.384367
PECAM1	CD47	-0.08302	0.412698	0.641	0.384367
PTPRC	CXCL9	0.417847	0.412698	0.641	0.384367
PDCD1LG2	IDO1	0.1629	0.412698	0.641	0.384367
ARG1	LAG3	-0.1478	0.555556	0.779915	0.255273
CD276	CD8A	-0.01909	0.555556	0.779915	0.255273
CD40LG	HLA-E	-0.22201	0.555556	0.779915	0.255273
IFNG	IL15	-0.14097	0.555556	0.779915	0.255273
CCL5	HAVCR2	0.159287	0.555556	0.779915	0.255273
CD68	CD276	-0.13276	0.730159	0.903417	0.136583
CD44	IL6	-0.23631	0.730159	0.903417	0.136583
LY6E	HLA-DQ	-0.1072	0.730159	0.903417	0.136583
MKI67	IFNG	-0.13271	0.730159	0.903417	0.136583
CSF1R	STAT2	0.105202	0.730159	0.903417	0.136583
VEGFA	PTPRC	-0.18274	0.730159	0.903417	0.136583
IDO1	CSF1R	0.115051	0.730159	0.903417	0.136583
LAG3	TIGIT	-0.12234	0.904762	0.957212	0.043466
VSIR	EPCAM	-0.17514	0.904762	0.957212	0.043466
ITGB2	CD86	-0.03456	0.904762	0.957212	0.043466
HLA-E	FOXP3	-0.05913	0.904762	0.957212	0.043466
CD274	PECAM1	-0.00804	0.904762	0.957212	0.043466
IL15	CMKLR1	0.03078	0.904762	0.957212	0.043466
HLA-DQ	IFNAR1	0.121909	0.904762	0.957212	0.043466
CD74	ITGB8	0.005247	0.904762	0.957212	0.043466
PSMB10	ARG1	0.007909	0.904762	0.957212	0.043466
HLA-DRB	CD40	0.038592	0.904762	0.957212	0.043466
B2M	ITGAM	-0.05696	1	1	0
STAT1	pan-melanocyte	0.004709	1	1	0
CD47	KRT	0.060056	1	1	0
IFNGR1	PDCD1LG2	-0.14095	1	1	0

Supplementary Table 5
